# Thermally Cured Gelatin-Methacryloyl Hydrogels Form Mechanically Modulating Platforms for Cell Studies

**DOI:** 10.1021/acs.biomac.5c00518

**Published:** 2025-07-17

**Authors:** Sara Lipari, Andrea Marfoglia, Giovanni Sorrentino, Sophie Cazalbou, Ludovic Pilloux, Pasquale Sacco, Ivan Donati

**Affiliations:** † Department of Life Sciences, University of Trieste, Via Licio Giorgieri 5, I-34127 Trieste, Italy; ‡ CIRIMAT, Université de Toulouse, CNRS, Toulouse INP, 35 Chemin des Maraichers, 31062 Cedex 09 Toulouse, France; § Laboratoire de Génie Chimique, Université de Toulouse, CNRS, Toulouse INP, 35 Chemin des Maraichers, 31062 Cedex 09 Toulouse, France; ∥ Department of Medical, Surgical and Health Sciences, University of Trieste, Strada di Fiume 477, 34139 Trieste, Italy; ⊥ International Centre for Genetic Engineering and Biotechnology (ICGEB), Area Science Park-Padriciano, 34149 Trieste, Italy

## Abstract

Gelatin methacryloyl (GelMA) is a polypeptide derived from the methacryloylation of gelatin and retains the thermoresponsive behavior of gelatin. When cooled, GelMA undergoes a sol–gel transition. By photo-cross-linking GelMA in a heated (“Hot”) or cooled (“Cold”) state, it results in a set of hydrogels with distinct properties. To date, the mechanical properties of these resulting hydrogels have not been fully elucidated. Here, we show that “Cold” hydrogels are viscoelastic, while “Hot” hydrogels are almost completely elastic. These features have remarkable implications for cell–substrate interactions *in vitro*: here, we show that fibroblasts, when cultured on these different substrates, adhere preferentially to Cold hydrogels. These results suggest that efficient cell adhesion requires specific mechanical properties of the substrate. This novel platform enables the precise control of different mechanical properties of GelMA by simply adjusting the cross-linking temperature, providing a flexible approach for the design of biologically inspired microenvironments *in vitro*.

## Graphical abstract

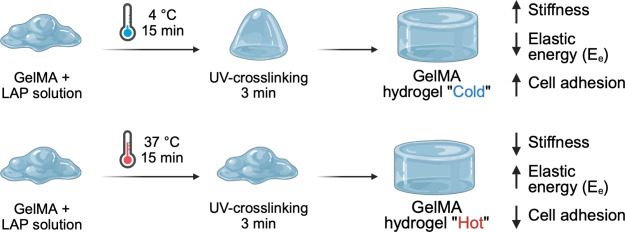

## Introduction

Gelatin is a polypeptide obtained from the partial hydrolysis of collagen.^1^ It became an attractive biomaterial due to its biocompatibility and biodegradability. Gelatin can provide cell adhesion arginine-glycine-aspartic acid (RGD) motifs,^2^ recognized by cells’ integrins and crucial for cell anchoring and spreading. Additionally, matrix metalloproteinases (MMPs) motifs are present,^3^ which are pivotal for cell matrix remodeling. Gelatin can form thermo-reversible hydrogels. At low temperatures, gelatin undergoes a conformational change from a coiled polymer to a helical, collagen-like structure.^1^ However, these gels lose their structural integrity and revert to a liquid state at physiological temperature (*T* = 37 °C).^4^

For this reason, different strategies to obtain stable hydrogels at physiological temperatures have been developed. One of the most explored is the methacrylation of gelatin lysine and hydroxylysine groups to obtain gelatin methacryloyl (GelMA), which can be cured by visible and UV light with the addition of a photoinitiator.^5^ GelMA was first described by Van Den Bulcke et al.^6^ and, since then, gained considerable attention as a biomaterial.^7–10^ GelMA, like gelatin, possesses RGD^11^ and MMP^12^ motifs. Another similarity with gelatin is GelMA’s capability of forming physical hydrogels when cooled, forming triple helical structures akin to those of collagen thanks to the presence of (Gly–X–Y)_*n*_ repeating sequences.^13–15^ Unlike gelatin, GelMA hydrogels can maintain their favorable physical properties at 37 °C due to irreversible covalent cross-linking. The mechanical and physical properties of GelMA can be tuned using different strategies such as the photoinitiator type and its concentration, the degree of substitution with methacrylate moieties, and/or the nature of gelatin.^16,17^ However, GelMA hydrogels exhibit an almost purely elastic nature and fail to replicate the viscoelastic properties of native extracellular matrices (ECMs).^18,19^ Different strategies have been proposed to modulate the mechanics of GelMA hydrogels,^20–24^ generally based on combination of gelatin with other components, such as alginate, to enhance the viscoelasticity of the hydrogels.

Here, we exploit the thermoresponsive nature of GelMA to modulate the viscoelasticity of the hydrogels.^25^ By performing the photocuring at either low (4 °C) or physiologically relevant (37 °C) temperatures, Cold or Hot hydrogels are obtained, respectively. In the present study, we report that the resulting hydrogels display different rheological properties, with a significant impact not only on the shear modulus (stiffness) but also on their viscoelastic features. Then, we employed this set of hydrogels as a substrate for 2D cell adhesion experiments. Compared to other hydrogel systems, such as those based on polyacrylamide, alginate, agarose, or chitosan and its lactose-modified derivative,^26–29^ GelMA has intrinsic RGD-presenting sites for cell adhesion without the need for chemical coupling or physical adsorption of additional adhesive sequences. This allowed us to gather evidence of the importance of these motifs in relation to the mechanics of the substrate.

## Materials and Methods

### Gelatin Methacryloyl (GelMA) Synthesis

The detailed experimental procedure has been previously described.^5^ Briefly, type B gelatin derived from bovine skin (50–120 bloom, pI = 4.7–5.3, Sigma-Aldrich, Saint Louis, MO, USA) was dissolved in 0.25 M sodium carbonate/sodium bicarbonate buffer prepared by dissolving 7.95 g of sodium carbonate (Sigma-Aldrich, Saint Louis, MO, USA) and 14.65 g of sodium bicarbonate (Sigma-Aldrich, Saint Louis, MO, USA) in 1 L of distilled water, achieving a final concentration of 10% w/V of gelatin. The pH of the solution was initially adjusted to 9 using 5 M sodium hydroxide or 1 M hydrochloric acid. Once the gelatin was fully dissolved, methacrylic anhydride (MAA, Sigma-Aldrich, Saint Louis, MO, USA) was added at a ratio of 0.1 mL MAA/g of gelatin under magnetic stirring at 500 rpm. The reaction was carried out at 50 °C for 3 h, after which the pH was readjusted to 7.4 to stop the reaction. The product was then filtered, dialyzed, freeze-dried, and stored at 4 °C for future use.

### TNBS Assay

The degree of substitution (DS) was quantified using the TNBS assay as previously described.^5^ Briefly, GelMA and type B gelatin samples were dissolved separately at a concentration of 1.6 mg/mL in a 0.1 M sodium bicarbonate buffer. Each sample solution (0.5 mL) was mixed with an equal volume of 0.01% 2,4,6-trinitrobenzenesulfonic acid (TNBS) solution prepared in 0.1 M sodium bicarbonate buffer. The mixtures were incubated for 2 h. To terminate the reaction, 0.25 mL of 1 M hydrochloric acid and 0.5 mL of 10% w/V sodium dodecyl sulfate (Sigma-Aldrich, St. Louis, MO, USA) were added. The absorbance was then measured at 335 nm using a UV spectrophotometer (Ultrospec 2100 pro, Amersham Bioscience, Amersham, UK). A glycine standard curve was constructed by using solutions at concentrations of 0, 8, 16, and 32 μg/mL to determine the concentration of amino groups. The determined degree of substitution of GelMA was 88 ± 7% of the available amino groups.

### Circular Dichroism (CD) Spectroscopy

CD spectra of dilute GelMA solutions (0.1 mg/mL, deionized water as solvent) were recorded with a Jasco J-700 spectropolarimeter.^25^ A quartz cell of 1 cm optical path length was used, always using the following setup: scan rate of 20 nm/min; wavelength range of 215–280 nm. GelMA samples were kept at 37 °C (heated GelMA) or 4 °C (cooled GelMA) until they were placed into the quartz glass measuring cuvette and transferred directly to the CD spectrometer. CD values are expressed as molar ellipticity [Θ]. For calculation of [Θ], a mean molar mass per amino acid residue of 90.50 g/mol was considered based on the amino acid composition of the unmodified gelatin type B published by Sewald et al.^30^ For GelMA, we corrected the mean molar mass per amino acid residue due to inserted methacrylic groups and the result was 94.70 g/mol.

### Hydrogel Formation

Sterile GelMA powder was dissolved in sterile deionized water at *T* = 37 °C under magnetic stirring. After complete dissolution of the polymer, the photoinitiator lithium phenyl-2,4,6-trimethylbenzoylphosphinate (LAP, Sigma-Aldrich, Saint Louis, MO, USA) and d-mannitol (Sigma-Aldrich, Saint Louis, MO, USA) were added to the GelMA solution. The final concentrations of each component were GelMA 5% w/V, LAP 0.1% w/V, and d-mannitol 300 mM. To obtain the two different conditions, namely, GelMA hydrogels “Hot” and “Cold”, the GelMA solution was poured into two different plates. The plates were incubated at 37 or 4 °C for 15 min to form Hot and Cold hydrogels, respectively. After incubation, the plates were irradiated for 3 min using an UV LED lamp (λ = 365 nm, 35 W, Hobbyland) to allow for photo-cross-linking.

### Mechanical Characterization of GelMA Hydrogels

Rheological characterization of the GelMA hydrogel disks (20 mm in diameter and 1.4–2 mm thick) was performed by means of an HAAKE MARS III rheometer operating at *T* = 37 °C with cross-hatched parallel plate configuration. GelMA solution was prepared as previously described and subsequently poured into two Petri dishes (Ø = 35 mm). The Petri dishes were incubated at *T* = 37 or 4 °C for 15 min to form Hot and Cold hydrogels, respectively. After incubation, the dishes were irradiated for 3 min using an UV LED lamp (λ = 365 nm, 35 W, Hobbyland) to allow for photo-cross-linking. The gap between plates was adjusted by means of short stress sweep tests (frequency, *i.e.*, ν = 1 Hz; stress (τ) range = 1–5 Pa) until a constant *G*′ at all data points was reached. Mechanical spectra have been recorded by means of frequency sweep tests (τ = 1 Pa; ν = 0.01–100 Hz) at *T* = 4 or 37 °C. The linear viscoelastic range was determined by means of stress sweep tests consisting of measuring the elastic (*G*′) and viscous (*G*″) moduli variation with increasing shear stress (1 Pa < τ < 3000 Pa) at ν = 1 Hz and *T* = 37 °C. The viscoelastic properties of GelMA hydrogels were determined by stress–relaxation experiments performed at *T* = 37 °C. Stress–relaxation tests were performed by applying a constant strain of 10% (*i.e.*, γ = 0.1) for 4500 s and tracking the decay of the stress as a function of time. Creep tests were performed at *T* = 37 °C by applying a constant stress of τ = 10 Pa. The increase in strain was monitored for 600 s. Temperature sweeps were performed by decreasing the temperature from *T* = 37 °C to *T* = 4 °C at a controlled rate (−1 °C/min) measuring *G*′ and *G*″ moduli at a constant ν = 1 Hz. During all of the measurements, a glass bell was used as a solvent trap to prevent water evaporation from the hydrogels.

### Absorption Assay

To evaluate the medium absorbing capacity of the hydrogels, GelMA Hot and Cold hydrogels were prepared as described previously. Gels were cut by using an 8 mm diameter biopsy punch. Hydrogel initial weights were recorded, and then, hydrogels were transferred to a 12-well plate and incubated in Dulbecco’s modified Eagle’s medium (ratio 1:10 V/V hydrogel:medium) at *T* = 37 °C. Weight measurements were taken at 1, 3, 7, 14, and 21 days. Absorption/degradation of the hydrogels was determined via the following formula (eq 1):

1 $$\Delta Weight\;\left(\%\right)=\frac{\left(W_{t}\times 100\right)}{W_{0}}$$

where *W*_*t*_ is the weight recorded at a given time point and *W*_0_ is the initial weight.

### Cell Culture

Murine fibroblasts NIH/3T3 (ATCC CRL-1658) were cultured in Dulbecco’s modified Eagle’s Medium (DMEM) High Glucose with 0.584 g/L l-glutamine and 0.11 g/L sodium pyruvate (EuroClone, Italy) supplemented with 10% V/V heat-inactivated Fetal Bovine Serum (FBS, Cat. no. ECS0180L, Lot. no. EUS00Q0, EuroClone, Italy) and 1% penicillin/streptomycin (EuroClone, Italy) in a humidified atmosphere of 5% CO_2_ at 37 °C. Primary Human Cardiac Fibroblasts (HCF) (PromoCell, Germany, Cat. No. C-12375) were cultured in Fibroblast Growth Medium 3 (PromoCell, Germany) supplemented with 10% V/V premixed supplements (PromoCell, Germany) and 1% penicillin/streptomycin (EuroClone, Italy) in a humidified atmosphere of 5% CO_2_ at 37 °C.

### Cell Seeding Atop GelMA Hydrogels (2D Substrates)

After the preparation of the GelMA solution, 80 μL of GelMA solution was poured in two different 48-wells plates. The two plates were incubated at 37 or 4 °C for 15 min to form Hot and Cold hydrogels, respectively. After incubation, the plates were irradiated for 3 min using an UV LED lamp (λ = 365 nm, 35 W, Hobbyland) to allow for photo-cross-linking. Cells were finally plated at a density of 30,000 cells/well with 320 μL of DMEM cell culture medium/well supplemented with 10% V/V or 0% V/V FBS. Further experiments were carried out with GelMA Hot and Cold hydrogels on which HCF cells were plated using a DMEM cell culture medium without FBS but supplemented with fibronectin (10 μg/mL, Sigma-Aldrich, Saint Louis, MO, USA) and bovine serum albumin (BSA, 3 mg/mL, Sigma-Aldrich, Saint Louis, MO, USA).

### Assessment of Cell Adhesion

After 24 h of incubation, cell culture medium was discarded and hydrogels were washed with PBS to remove nonadherent cells. Then, 300 μL/well of PBS was added and the number of adherent cells/mm^2^ was quantified by Fiji-ImageJ software (multipoint tool) from images acquired through a Nikon Ti Eclipse inverted bright-field microscope equipped with an Intensilight Epi-fluorescence Illuminator and a Plan Fluor 10× DIC L N1 objective. Acquisition was performed with a DS-Qi2 16 Mpixel camera (Nikon).

### Statistical Analysis and Software

Statistical comparisons and graphical elaborations were carried out using GraphPad Prism software. The data distributions were tested for normality using the D’Agostino-Pearson omnibus or the Shapiro–Wilk normality test. Parametric or nonparametric tests were then selected to perform the statistics between the different groups. *N* represents the number of independent experiments, and *n* represents the number of technical replicates. The specific statistics are reported as indicated in the figure captions.

## Results and Discussion

Gelatin methacryloyl (GelMA) was prepared by treating the protein with methacrylic anhydride at a slightly basic pH. Approximately 88% of the available amino groups in gelatin were modified with methacrylate moieties. Despite the high degree of substitution, the synthesized GelMA retains the ability to form collagen-like triple helix structures upon cooling, albeit only to a limited extent,^31^ as indicated by the weak positive CD peak at around 221 nm (Figure S1, Supporting Information). When the same macromolecule is heated, the positive peak disappears and the CD signal appears completely negative in the 215–260 nm range. It should be noted that both heated and cooled GelMA samples show a negative band at around 238 nm, which has been correlated with the formation of single helices,^25,32^ with a more negative molar ellipticity for the heated than for the cooled sample. Hence, the disentangling of triple-helical structures comes with a higher number of single helices in GelMA. At a concentration of 5% w/V, GelMA showed a thermoresponsive behavior, switching from a liquid state at high temperatures to a gel state at low temperatures,^33–35^ thus confirming the formation of triple helix structures as already detected by CD.

We cross-linked GelMA through a photo-cross-linking of the methacrylate moieties using two approaches (Figure 1A). In the first one, GelMA was photoreticulated in liquid state at *T* = 37 °C and the hydrogel was referred to as “Hot”. In this case, the temperature prevents the formation of triple helix structures while some single helices are present in the three-dimensional network. In the second approach, GelMA solution was cooled to *T* = 4 °C allowing a physical gel formation. This was followed by a photoreticulation of the methacrylate moieties resulting in a hydrogel that was referred to as “Cold”. Both Hot and Cold hydrogels were characterized through rheometry. As reported by other authors, photo-cross-linking of GelMA at 4 °C allows one to tune its mechanical properties and, in general terms and irrespective of the formation of a physical hydrogel, leads to stiffer networks.^25,36,37^

**Figure 1:**
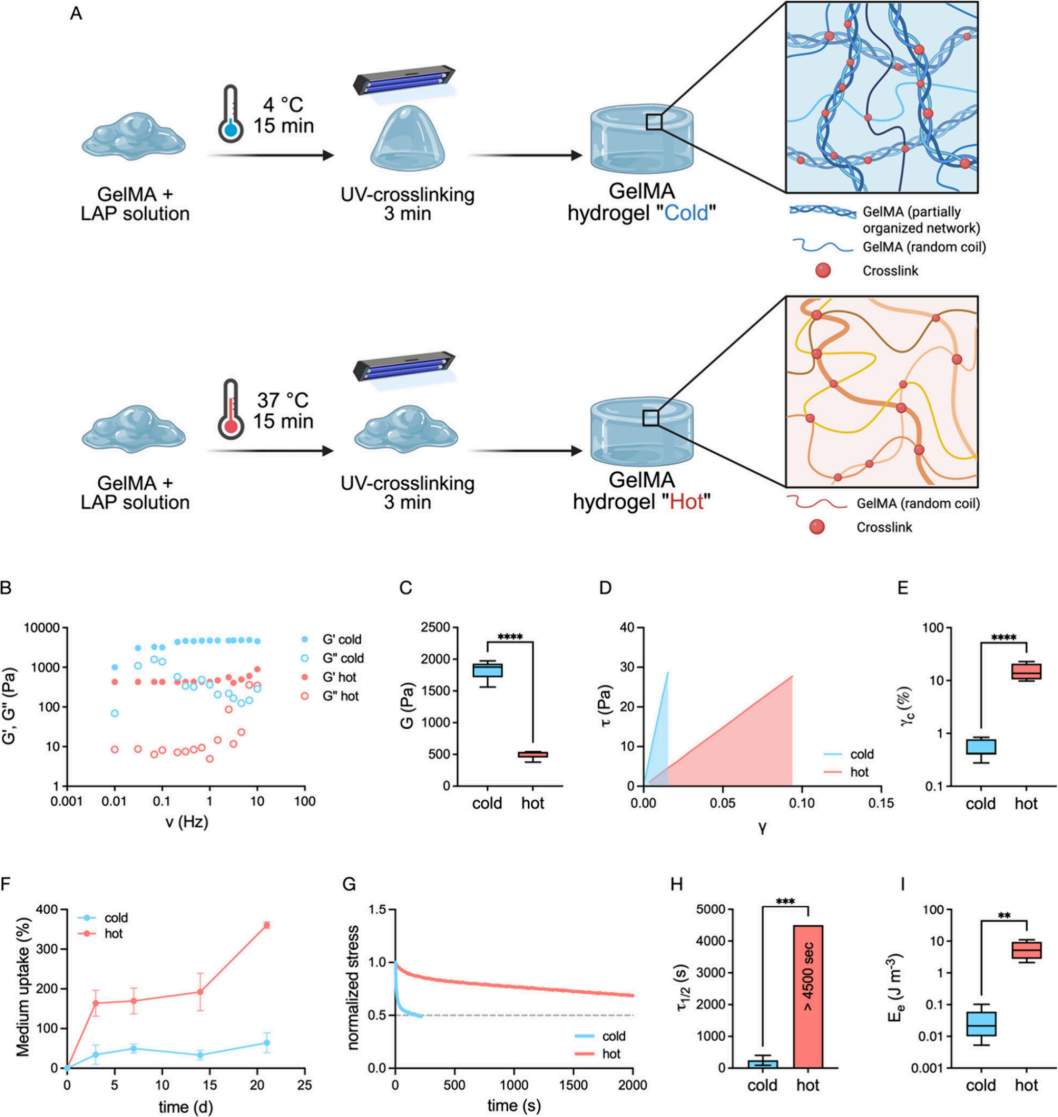
Elastic/viscoelastic transition of GelMA hydrogels is governed by the cross-linking temperature. (A) Schematic cartoon showing the methodology used for the generation of the different GelMA hydrogels. Created in BioRender. Sacco, P. (2025) https://BioRender.com/zgimu0u. (B) Mechanical spectra showing the dependence of *G*′ and *G*″ moduli (Pa) on frequency (Hz) at *T* = 37 °C. (C) Shear modulus *G* of the GelMA hydrogels. Data obtained from averaging (mean ± s.d.; *n* = 6) the shear modulus from mechanical spectra recorded at *T* = 37 °C (Appendix 1, SI) (****p < 0.0001 by two-tailed *t* test). (D) Shear stress–strain curve profile of GelMA hydrogels at *T* = 37 °C. Solid light red or blue areas were filled to emphasize the extension of the linear stress–strain (elastic) region. (E) Critical strain, γ_c_, for the GelMA hydrogels. Data obtained from averaging (mean ± s.d.; *n* = 5–7) the data from long stress sweep at *T* = 37 °C (Appendix 2, SI) (****p < 0.0001 by two-tailed *t* test). (F) Medium uptake experiments performed on GelMA hydrogels incubated in DMEM High Glucose cell culture medium at *T* = 37 °C and 5% CO_2_ over 21 days (mean ± s.d.; *n* = 5). Data are expressed as weight change in percentage with respect to *t* = 0 (*i.e.*, weight of dry samples). (G) Normalized stress over time derived from stress–relaxation measurements. The gray dotted line guides the eye to the value corresponding to half of the normalized stress, corresponding to the τ_1/2_ value (*i.e.*, time needed to relax the stress to half of the initial value). (H) τ_1/2_ values for the GelMA hydrogels. Data obtained from averaging (mean ± s.d.; *n* = 6) the τ_1/2_ values derived from stress–relaxation tests at *T* = 37 °C (***p < 0.001 by two-tailed *t* test). (I) Elastic energy for the GelMA hydrogels. Data obtained from averaging (mean ± s.d.; *n* = 5–7) the data from long stress sweep at *T* = 37 °C (**p < 0.01 by two-tailed *t* test).

First, the hydrogels were analyzed by recording the mechanical spectra of both Cold and Hot hydrogels at *T* = 37 °C. The Cold hydrogel shows both higher storage modulus (*G*′) and higher loss modulus (*G*″) than the Hot hydrogel at all frequencies analyzed (Figure 1B). Interestingly, *G*″ in the Cold hydrogel exhibits a peak around 0.06 Hz, which corresponds to a relaxation time of around 16 s. This profile seems to indicate weak, transient interchain interactions in the three-dimensional network.^38^ In nice agreement with previous studies,^36,39^ the calculated shear modulus, *G*, derived from data elaboration through the Maxwell model of the Cold hydrogel (∼2 kPa) is higher than that of the Hot one (∼0.5 kPa) (Figure 1C and Appendix 1, Supporting Information). It is noteworthy that these stiffness values correspond well with the elastic response of some biological soft tissues or reconstituted extracellular matrices.^40^ It has been proposed that the higher stiffness of Cold hydrogels is a result of the increased cross-linking density, facilitated by the closer distance between chains in the gel state.^37^ To provide information about the macromolecular structure of hydrogels, the latter were analyzed using the theory of rubber elasticity (Appendix 3, Supporting Information).^41^ The calculated average mesh size was smaller for the Cold hydrogel than for the Hot one. Therefore, a calculated average contour length between cross-links, *L*_C_, is shorter in the former than in the latter. By assuming a persistence length of the random coil (*l*_P_ = 2 nm^42^) for both Cold and Hot GelMA hydrogels, it holds *L*_C_ ≫ *l*_P_, thus supporting the use of the rubber elasticity theory (Appendix 3, Supporting Information).

Hot and Cold hydrogels were further investigated by performing a temperature sweep analysis (Figure S2, Supporting Information). The loss and storage moduli of GelMA hydrogels were monitored by gradually decreasing the temperature from 37 to 4 °C. When looking at the storage modulus values over the tested temperature range, two different behaviors are observed. For Cold hydrogels, the initial reduction in the temperature leads to a slight decrease in the elastic modulus, which is qualitatively consistent with the predictions of the rubber elasticity theory. When the temperature is lowered further, an upturn in *G′* can be observed, which indicates a minor effect of the formation of triple helices and their bundling. Cold hydrogels were indeed formed with the triple helix structures already present. The high photochemical reticulation freezes the existing network and does not allow for extended formation of bundles with a substantial improvement in mechanical performance upon decreasing the temperature. A different pattern was found for Hot hydrogels: the lower photo-cross-linking density enables the formation of triple helix structures and their bundling, which leads to an increase in shear modulus when the temperature is lowered.

The linear stress–strain, *i.e.*, elastic, region of the hydrogels was investigated by stress sweep experiments at *T* = 37 °C. Interestingly, we report that Hot hydrogels display a more extended elastic response compared to their Cold counterparts, reflecting a higher elastic behavior (Figure 1D and Figure S3, Supporting Information). The comparison between the tanδ (*i.e.*, *G*″/*G*′ at *ν* = 1 Hz) values derived from the frequency sweeps corroborate the greater elastic behavior for the Hot hydrogels (Figure S4, Supporting Information). This is additionally confirmed by the critical strain (γ_c_),^43^ namely, the deformation at which the hydrogels exit the linear region, which is significantly higher in Hot hydrogels compared to Cold counterpart (Figure 1E and Appendix 2, Supporting Information).

Hot and Cold hydrogels present different medium absorption behaviors that are dependent on the different network organizations. While Hot hydrogels increase up to ∼350% of their initial weight over 21 days, with a particularly large gain in mass after 15 days, Cold hydrogels increase by only ∼50% (Figure 1F). The random coil-like network organization of Hot hydrogels appears to be looser and more prone to absorb water compared to the network of Cold hydrogels, which is less likely to rearrange. The swelling equilibrium of ideal networks reflects a balance between the elastic pressure, given by the stretching of the polymer chains between cross-links and the osmotic pressure acting to swell the three-dimensional structure. Following the approach of Flory-Rehner^44^ and considering the end-to-end distance of the polymer strand in the hydrogel preparation state as (eq 2):

2 $$\langle {h_{0}}^{2}\rangle =N_{K}{l_{K}}^{2}$$

where *l*_K_ and *N*_K_ are the length and the number of the Kuhn’s segments, respectively, the swelling equilibrium, *Q*_e_, at low salt concentration^45^ can be written as (eq 3):

3 $$Q_{e}∼{N_{K}}^{2}\alpha^{3/2}$$

Given the lower cross-linking density and hence the higher number of Kuhn’s statistical segments in the elastic polymer chains (Table S1, Appendix 3, Supporting Information), a higher swelling is expected for the Hot GelMA with respect to the Cold sample. In particular, by assuming the same ionization degree, α, for both the Cold and Hot hydrogels, the ratio between the swelling equilibrium of the two systems becomes (Table S1, Appendix 3, Supporting Information) (eq 4):

4 $$\frac{Q_{e,hot}}{Q_{e,cold}}∼\frac{{\left(N_{K,hot}\right)}^{2}}{{\left(N_{K,cold}\right)}^{2}}∼8.2$$

The application of the rubber elasticity and the Flory–Rehner theory to biopolymer hydrogels undoubtedly poses some questions. In particular, while for the Hot sample the photoreticulation leads to a chemical hydrogel where the “quasi” point-like cross-links conform quite well to the rubber elasticity theory, in the Cold sample, the situation is more complicated. Indeed, the presence of both chemical cross-links and physical reticulations might represent a serious hurdle to the application of the rubber elasticity, and hence, the results should be considered with caution. However, the reasonable agreement between results calculated on the basis of eq 4 and the experimental data of Figure 1F (*i.e.*, approximately 7) is quite reassuring. An additional limitation of the present approach (Appendix 3, Supporting Information) relies on using the same value of *l*_p_ for both Hot and Cold hydrogels. All in all, while rubber elasticity could provide comparative indications on the behavior of biopolymer-based hydrogels obtained under similar experimental conditions, one should restrain from excessive speculations.

Then, we undertook stress–relaxation experiments to provide information about the viscoelastic properties of the hydrogels. Indeed, while other authors have extensively described the increase in stiffness of physically and chemically cross-linked GelMA hydrogels, to the best of our knowledge, there is a lack of information whether this cross-linking strategy affects the viscoelastic properties of the resulting hydrogel.^46^ The experiments on stress–relaxation showed that the Hot hydrogels were almost purely elastic, while the Cold hydrogels dampened the stress over time (Figure 1G). While the τ_1/2_ (*i.e.*, time needed to relax the stress to half of the initial value^47^) values of Hot GelMA were large (>4500 s), the Cold hydrogels relaxed the stress in less than 500 s (Figure 1H). It is important to recall that the stress–relaxation experiments were performed at a constant strain of 10% relevant for cell activities,^48^ which falls in the linear stress–strain range for the Hot hydrogel system and in the nonlinear range for the Cold counterpart. To exclude the possibility that this different mechanical behavior is due to different mechanical stimulation, we performed creep experiments as a control, applying a very low stress, namely, 10 Pa, which is within the linear stress–strain range for both investigated systems (Figure 1D). Even in this case, we found a very high viscoelastic behavior for the Cold hydrogels and an almost purely elastic response for the Hot counterpart (Figure S5, Supporting Information), in agreement with the stress–relaxation data. Therefore, the Cold GelMA hydrogels are not only stiffer but also much more viscoelastic than the Hot hydrogels. In addition, elastic energy (*E*_e_) has recently emerged as a relevant mechanical property that describes how much energy is stored in the linear region of a material before plastic deformation occurs, which extends from γ = 0 to γ_c_ (eq 5), where γ_c_ (critical strain) refers to the onset of the plastic deformation (Appendix 2, Supporting Information):^49^

5 $$E_{e}=\int_{\gamma =0}^{\gamma_{c}}\left({\frac{d\sigma }{d\gamma }|}_{\gamma =0}\right)\gamma \;d\gamma =\frac{1}{2}G_{0}{\gamma_{c}}^{2}$$

where *G*_0_ is the initial shear modulus of the hydrogel. *E*_e_ describes how much energy is required to enter the nonlinear stress–strain region of a material and thus enter the region of plastic deformation. The elastic energy is influenced by both stiffness (shear modulus) and critical strain in a complex manner. Indeed, stiff but brittle hydrogels, despite a high shear modulus, show a very low value of the critical strain and hence an overall low elastic energy. This implies a limited linear stress–strain response and a hydrogel showing plastic deformation. At variance, for soft hydrogels, the shear modulus is very low. However, the value of the critical strain could be quite extended, leading to elastic hydrogels with high elastic energy, *E*_e_.^49^

In good agreement with the stress–relaxation measurements, the Hot hydrogels show a significantly higher *E*_e_ than the Cold hydrogels, indicating that the plastic deformation of the latter is triggered at a much lower energy threshold (Figure 1I). The difference in viscoelastic behavior is an additional indication that the macromolecular arrangement of biopolymer chains in the Hot and Cold hydrogels differs drastically. More specifically, the viscoelastic nature of collagen, which can dissipate stress by fiber reorganization, is somehow preserved in the Cold hydrogel.^40,50^ The calculated *E*_e_ agrees well with the stress–relaxation behavior and underlines the viscoelastic nature of the Cold hydrogels, in contrast to the almost purely elastic Hot hydrogels. Altogether, these results describe how the Hot/Cold strategy for GelMA hydrogel formation can be employed to obtain either soft and elastic hydrogels (Hot) or stiff and viscoelastic hydrogels (Cold).

Given the strong mechanical differences between Hot and Cold hydrogels, we decided to use them as substrates to explore cell adhesion behavior. For this purpose, we used either murine fibroblasts (NIH/3T3) or primary human cardiac fibroblasts (HCF). In view of the presence of RGD domains on both Hot and Cold GelMA hydrogels, we started focusing on the adhesion of cells using culture medium completely devoid of Fetal Bovine Serum (FBS).

Adhesion was observed in both cell lines (Figure 2A,B). In a previous work, hydrogels containing neither RGD peptides nor protein-binding motifs showed a threshold value of 0.15 J/m^3^ for elastic energy, above which no cell adhesion occurred.^49^ The present work shows that the threshold, if present, shifts to higher values of elastic energy when a hydrogel containing binding motif is used. While the elastic energy of the Cold hydrogel is slightly below 0.1 J/m^3^, *i.e.*, well below the threshold previously indicated for the Hot hydrogel, cell adhesion occurs even at an elastic energy of almost 10 J/m^3^. However, it should be noted that the number of adherent cells decreases upon increasing elastic energy of the substrate, which is consistent with a previous report.^49^ Besides a threshold allowing for cell adhesion, cell lines show different sensitivity to elastic energy variations of the substrate.^49^ This is seen also in the present case comparing the effect of the substrate on NIH/3T3 and on HCF. In fact, a slight difference in the number of adherent cells was noticed for the first cell line when comparing Cold, *i.e.*, with lower elastic energy, to Hot, *i.e.*, with higher elastic energy, hydrogels. At variance, the number of adherent cells is not influenced in the case of HCF. Additionally, a comment on the sensitivity of the cell lines to the elastic properties of the substrate can be made based on the overall number of adherent cells. For both Cold and Hot hydrogels, the number of adherent NIH/3T3 overcomes that of HCF; this might be due to their different capacities to deform the substrate, although this needs to be further investigated.

**Figure 2:**
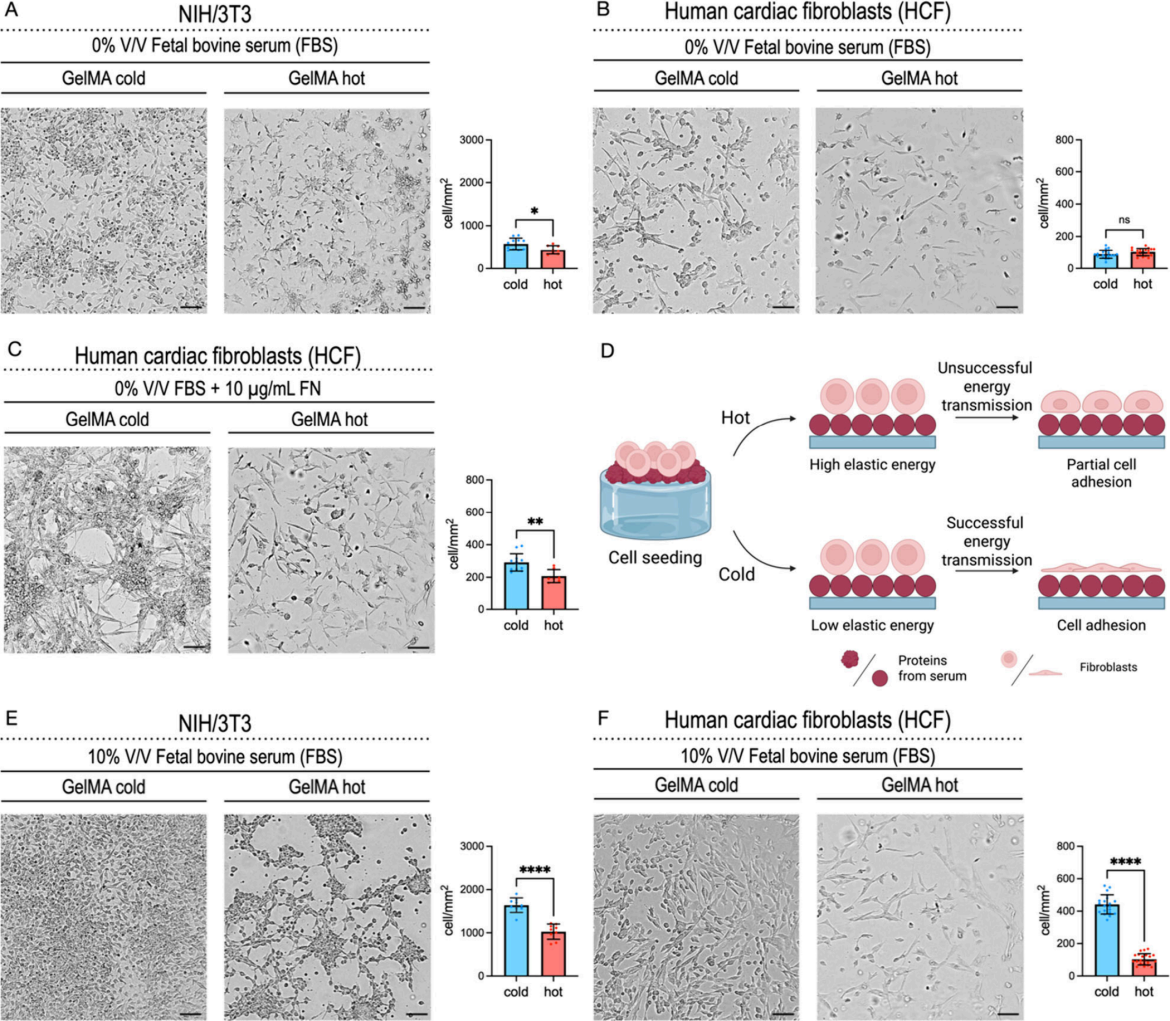
Cell adhesion on GelMA Hot and Cold hydrogels is governed by different mechanics and cell binding sites. (A) Images from cell adhesion test of NIH/3T3 after 24 h from seeding (30,000 cells/well) atop GelMA hydrogels. Cells were cultured in the presence of 0% V/V FBS in cell culture medium. Number of NIH/3T3 cells per mm^2^ adhering on GelMA hydrogels was averaged and expressed as mean ± s.d. (*n* = 5, *N* = 3) (*p < 0.05 by two-tailed *t* test). (B) Images from cell adhesion test of HCF after 24 h from seeding (30,000 cell/well) atop GelMA hydrogels. Cells were cultured in the presence of 0% V/V FBS in cell culture medium. Number of HCF cells per mm^2^ adhering on GelMA hydrogels was averaged and expressed as mean ± s.d. (*n* = 5, *N* = 3) (ns = not significant by a two-tailed *t* test). (C) Images from cell adhesion test of HCF after 24 h from seeding (30,000 cell/well) atop GelMA hydrogels. Cells were cultured in the presence of 0% V/V FBS + 10 μg/mL of fibronectin (FN) + 3 mg/mL bovine serum albumin (BSA) in cell culture medium. Number of HCF cells per mm^2^ adhering on GelMA hydrogels was averaged and expressed as mean ± s.d. (*n* = 5, *N* = 3) (ns = not significant by two-tailed *t* test). (D) Schematic representation of the proposed cell adhesion mechanism on top of GelMA Hot and Cold hydrogels. Created in BioRender. Sacco, P. (2025) https://BioRender.com/b69d812. (E) Images from cell adhesion test of NIH/3T3 after 24 h from seeding (30,000 cell/well) atop GelMA hydrogels. Cells were cultured in the presence of 10% V/V FBS in a cell culture medium. Number of NIH/3T3 cells per mm^2^ adhering on GelMA hydrogels was averaged and expressed as mean ± s.d. (*n* = 5, *N* = 3) (****p < 0.0001 by two-tailed *t* test). (F) Images from cell adhesion test of human cardiac fibroblasts (HCF) after 24 h from seeding (30,000 cell/well) atop GelMA hydrogels. Cells were cultured in the presence of 10% V/V FBS in cell culture medium. Number of HCF cells per mm^2^ adhering on GelMA hydrogels was averaged and expressed as mean ± s.d. (*n* = 5, *N* = 3) (****p < 0.0001 by two-tailed *t* test).

We next investigated the effect on cell adhesion of implementing the density of the binding motifs by supplementing the medium with fibronectin (FN). It is well-known that fibronectin can enhance cell adhesion due to changes in its three-dimensional conformation and exposure of the synergy site enhancing actomyosin contractility.^51^ Figure 2C shows that the presence of fibronectin in the medium enhances the overall HCF cell adhesion for both Hot and Cold hydrogels. In addition, compared to the nonsupplemented condition, the role of elastic energies of the substrate in cell adhesion becomes evident. In fact, while in nonsupplemented serum, the number of adherent HCF atop the hydrogel was the same irrespective of the preparation process of the hydrogel, in the presence of fibronectin, there is a marked increase of cell adhesion on the substrate showing the lowest elastic energy.

The results of these experiments seem to indicate that, despite the presence of RGD motifs in GelMA, proteins that exhibit cell adhesion motifs are crucial to further enhance cell adhesion to substrates. We hypothesize here that these proteins are also critical mediators of energy transfer and that elastic energy is a crucial player in energy transmission on the substrate (Figure 2D). We reported how cell adhesion correlates with at least two factors: (1) a relatively high presence of integrin-binding sites, hence exhibiting high anchoring point density; (2) a substrate requiring a relatively high viscoelasticity, in this manuscript reported as low elastic energy, needed to access the plastic region early. Indeed, the addition of fibronectin plays two roles: (i) enhances the number of adherent cells to the substrate in general terms and (ii) exacerbates the role of the elastic energy.

To test this hypothesis, we used a culture medium enriched with 10% V/V FBS. Piazza et al. recently reported on the importance of serum for cell adhesion to agarose hydrogels.^52^ Similarly, we investigated the effect of serum on cell adhesion on the Hot and Cold GelMA hydrogel. Both the NIH/3T3 and HCF cells were plated on Cold or Hot GelMA substrates in serum-enriched medium (Figure 2E,F). Fetal Bovine Serum (FBS) contains key adhesive proteins, of which fibronectin and vitronectin are the most abundant,^53,54^ so it is expected that its presence increases the number of adherent cells. This is seen from the comparison between Figure 2E,F. For both NIH/3T3 and HCF the number of adherent cells doubles when the medium is supplemented with FBS with respect to the FBS-free medium. In general terms, a correlation was found between the number of adhering cells to the substrate with the stiffness and viscoelasticity of the hydrogels; that is, stiffer, stress-relaxing hydrogels with low elastic energy significantly favor cell adhesion (Cold hydrogels). This result is in good agreement with earlier studies on this topic, which were carried out either with agaroses of different origins or with collagen-coated polyacrylamide hydrogels.^29,55^ Moreover, the addition of FBS stresses the role of elastic energy in the overall cell adhesion. In fact, the change in the elastic energy of the substrate leads to a stronger effect on cell adhesion when FBS is present.

Fibronectin and other adhesion proteins are important mediators in the energy transfer from the cell to the substrate. When the elastic energy is high, the integrins of the cells bind to the proteins atop the hydrogels and try to yield the substrate via the actomyosin-integrin axis, creating a chain of energy transfer between cell, protein, and substrate. However, the high elastic energy prevents the successful transmission of traction forces and thus plastic deformation to a certain extent. If, on the other hand, the elastic energy of the substrate is low, then the energy transfer via the cell–protein–substrate axis leads to successful adhesion and spreading. These considerations are consistent with earlier findings in which traction forces generated by cells on substrates with low and high elastic energy were determined. Indeed, the calculated maximum traction forces were around 200 and 20 Pa for low and high elastic energy substrates, respectively, meaning that cells access the plastic region of the substrate easily in the former case.^49^ Although the contributions of stiffness and viscoelasticity cannot be decoupled in this set of GelMA hydrogels, the overall results suggest a critical balance between the mechanical transmission of cellular forces and the consequent transduction of biophysical cues. Additional information on the exact cellular machinery is required to draw final conclusions on the molecular mechanism orchestrating cell adhesion on GelMA hydrogels.

## Conclusions

In the present work, we have characterized the mechanical properties of GelMA hydrogels cross-linked by either photo-cross-linking (Hot) or physical followed by photo-cross-linking (Cold). While it was already known that the latter cross-linking strategy increases the stiffness of the resulting hydrogels, we unveil that also the viscoelasticity can be modulated. Indeed, the Cold hydrogels are stress relaxing and present a different stress–strain response compared to Hot hydrogels, which are almost purely elastic. Previously, the mechanical differences between photo- and physical + photo-cross-linked hydrogels were attributed to an increased cross-linking density. However, given this new insight into their viscoelastic behavior, we suggest that the differences are a result of a different network conformation, somewhat recapitulating that of collagen, which is reconstituted in the Cold network.

We then investigated the different cell adhesion properties of the Hot and Cold hydrogels with murine and human cardiac fibroblasts. Previous work carried out with agarose has shown that cell adhesion correlates inversely with the elastic energy of a substrate. Unlike agarose, GelMA hydrogels possess intrinsic cell adhesion motifs, *i.e.*, the RGD motif, so that the chemical functionalization of the substrate is unnecessary. We found minimal differences in cell adhesion between Hot and Cold hydrogels when the serum-derived adhesive proteins are removed from the culture medium. The differences in cell adhesion between the two hydrogels were emphasized by increasing the anchoring points by adding fibronectin to the medium. This revealed the role of elastic energy, as the number of adherent cells was higher on stiff and viscoelastic hydrogels (Cold). Finally, when the medium was supplemented with 10% V/V FBS, a large and clear difference was observed between the adherent cells on substrates with low (Cold) and high (Hot) elastic energy. Similar to agarose, substrates exhibiting low elastic energy were more favorable for cell adhesion than substrates exhibiting high elastic energy. In summary, we conclude that adhesive proteins in serum, such as fibronectin, in combination with the elastic energy of the substrate, are important mediators in the interaction between cells and substrates that can enhance the biological response to intrinsic RGD motifs of GelMA substrates.

## Supplementary Material



## Data Availability

Raw data generated during this study are available upon reasonable request. Correspondence and requests for materials should be addressed to S.L.

## Notes

Additional figures; Appendix 1, which contains the analysis of the mechanical spectra for GelMA Hot and Cold hydrogels using the Maxwell model; Appendix 2, which contains the analysis of the long stress sweep data for GelMA hydrogels and the determination of the critical deformation at which the nonlinear stress–strain region begins; Appendix 3, which contains the rubber elasticity theory for the calculation of the mesh size and contour length of GelMA Hot and Cold hydrogels (PDF)
