# Supplementary material for: Thermally Cured Gelatin-Methacryloyl Hydrogels Form Mechanically Modulating Platforms for Cell Studies
Source: Biomacromolecules. 2025 Jul 17;26(8):5086–95. doi: 10.1021/acs.biomac.5c00518 (PMC12344701; doi:10.1021/acs.biomac.5c00518)
Supplement: Supplementary file 1 [file bm5c00518_si_001.pdf]

Supporting Information

Thermally Cured Gelatin-Methacryloyl Hydrogels Form Mechanically Modulating Platforms for Cell Studies

Sara Lipari[1 # *], Andrea Marfoglia[2,3,4,5 #], Giovanni Sorrentino[4,5], Sophie Cazalbou[2], Ludovic Pilloux[3], Pasquale Sacco[1*], Ivan Donati[1].

[1] Department of Life Sciences, University of Trieste, Via Licio Giorgieri 5, I-34127 Trieste, Italy

[2] CIRIMAT, Université de Toulouse, CNRS, Toulouse INP, 35 Chemin des Maraichers, 31062 Toulouse Cedex 09 - France

[3] Laboratoire de Génie Chimique, Université de Toulouse, CNRS, Toulouse INP, 35 Chemin des Maraichers, 31062 Toulouse Cedex 09 - France

[4] Department of Medical, Surgical and Health Sciences, University of Trieste, Strada di Fiume 477, 34139, Trieste, Italy

[5] International Centre for Genetic Engineering and Biotechnology (ICGEB), Area Science Park-Padriciano, 34149 Trieste, Italy

[#] These authors contributed equally

[*] Corresponding authors: psacco@units.it; sara.lipari@phd.units.it

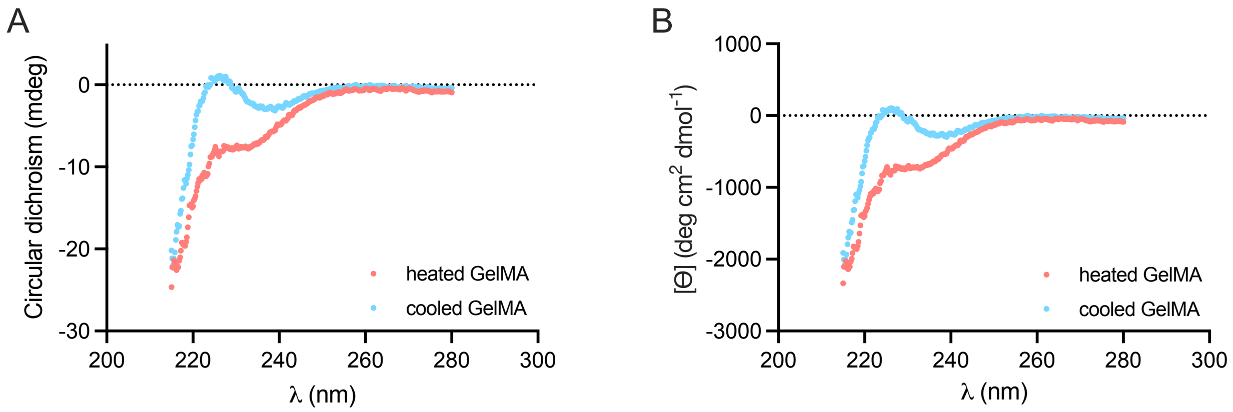

Figure S1. Circular dichroism expressed in mdeg (A) or molar ellipticity, [Θ] (B) of heated (37 °C) and cooled (4 °C) GelMA solutions (0.1 mg/mL, deionized water as solvent). The light blue spectrum was recorded after cooling the solution in a water-ice bath. The red spectrum was recorded after heating the previous solution.

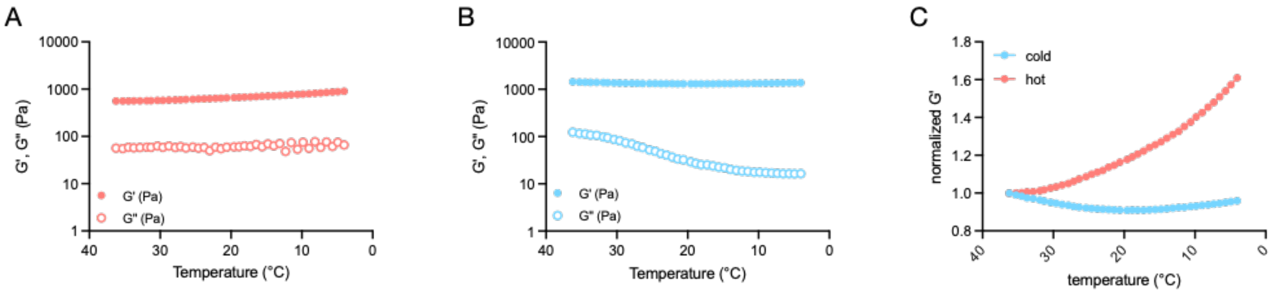

Figure S2. Temperature sweep performed from 37 °C to 4 °C for GelMA Hot (A) and Cold (B) hydrogels. (C) Normalized G' for Hot and Cold hydrogels derived from the temperature sweep tests performed gradually decreasing the temperature (1 °C/min) from 37 °C to 4 °C.

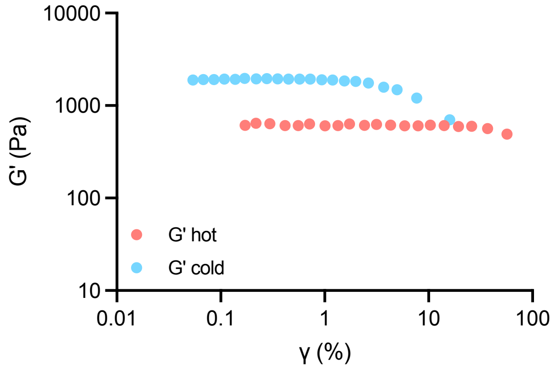

Figure S3. Storage modulus (G') profiles for Hot and Cold GelMA hydrogels as a function of the strain (γ in %).

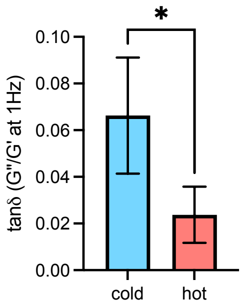

Figure S4. Tanδ values expressed as the ratio G''/G' at ν = 1 Hz. Data obtained from averaging (mean ± s.d.; n = 5) the values derived from frequency sweep at T = 37 °C (* $p < 0.05$ by two-tailed t test).

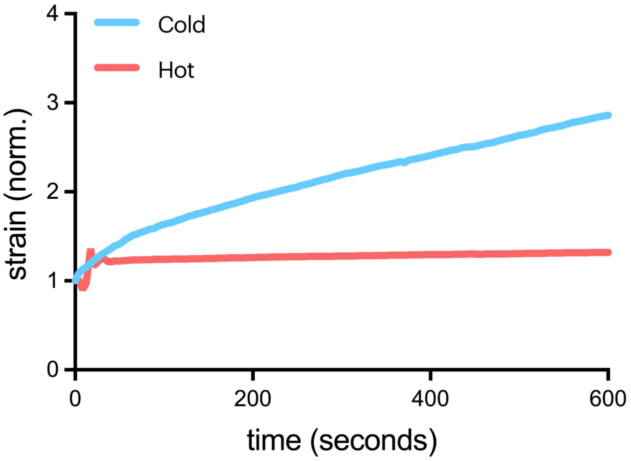

Figure S5. Representative creep profiles for Cold and Hot hydrogel systems. Data are reported as strain (norm.) as a function of time in agreement with literature ([1]).

Appendix 1. Analysis of the mechanical spectra for GelMA Hot and Cold hydrogels using the Maxwell model.

Mechanical spectra derived from frequency sweep tests of GelMA Hot and Cold hydrogels were interpreted in terms of a generalized Maxwell model composed of a sequence of elements in parallel (*i.e.* spring and dashpot) to which an additional spring has been added. The storage (G') and the loss (G'') moduli were fitted as a function of the pulsation ω (= 2πν where ν is the frequency used) according to the following equations (Equations S1 and S2) [2]:

$$G' = G_e + \sum_{i=1}^{n} \frac{G_i(\lambda_i\omega)^2}{1+(\lambda_i\omega)^2} \qquad \text{eq. S1}$$

$$G'' = \sum_{i=1}^{n} \frac{G_i(\lambda_i\omega)}{1+(\lambda_i\omega)^2} \qquad \text{eq. S2}$$

with

$$G_i = \frac{\eta_i}{\lambda_i}$$

where *n* represents the number of Maxwell elements considered while G_i, η_i, and λ_i represent the spring constant, the dashpot viscosity, and the relaxation time of the *i*-th Maxwell element, respectively. G_e is the spring constant of the last Maxwell element which is supposed to be purely elastic. The fitting of the experimental data was performed assuming that relaxation times are scaled by a factor 10. Hence, the parameters of the fitting are G_e, η_i, and λ_1. The number of the Maxwell elements was selected to minimize the product $\chi^2 N_p$, where χ^2 is the sum of the squared errors, while $N_p(= 2 + n)$ indicates the number of fitting parameters. For all the samples analyzed, the number of Maxwell elements resulted equal to 3. Figure S6 shows an example of the mechanical spectra of GelMA Hot and Cold hydrogels and the fitting of the experimental data using the generalized Maxwell model.

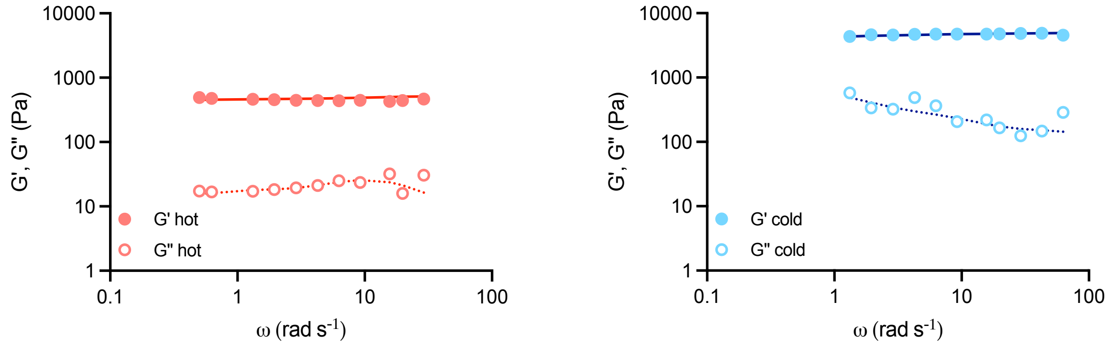

Figure S6. Dependence of storage (G', full dots) and loss (G'', empty dots) moduli from pulsation for GelMA hydrogels Hot and Cold. Solid and dotted lines represent the best-fit of the experimental data using equations S1 and S2.

Shear modulus, G, was then derived from the generalized Maxwell model, as (eq. S3):

$$G = G_e + \sum_{i=1}^{n} G_i \qquad \text{eq. S3}$$

<u>Appendix 2</u>. Analysis of the long stress sweep data for GelMA hydrogels and determination of shear strain and critical deformation,γ_c.

The different long stress sweep measurements for GelMA Hot and Cold hydrogels were modeled, from the phenomenological point of view, using eq. S4.

$$\sigma = \frac{G}{1+b\gamma}\gamma \qquad \text{eq. S4}$$

where σ represents the stress, γ is the strain while *G* and *b* are fitting parameters. *G* corresponds to the shear modulus at $\gamma_{\to 0}$ (eq. S5).

$$G = \lim_{\gamma \to 0} \frac{d\sigma}{d\gamma} \qquad \text{eq. S5}$$

The critical strain (γ_c), which marks the onset of the non-linear behavior, was defined, in line with previous works [3,4], as (eq. S6):

$$\left.\frac{d\sigma}{d\gamma}\right|_{\gamma=\gamma_c} = 0.95 \lim_{\gamma \to 0} \frac{d\sigma}{d\gamma} \qquad \text{eq. S6}$$

The equation S6 can be rewritten as (eq. S7):

$$G|_{\gamma_c} = 0.95\, G \qquad \text{eq. S7}$$

Which, combined with eq. S4, allowed determining the critical strain, as (eq. S8):

$$\frac{G}{1+b\gamma_c} = 0.95G \qquad \text{eq. S8}$$

Figure S7 shows an example of a long stress sweep for GelMA Cold hydrogel and the fitting of the experimental data using the equation S4.

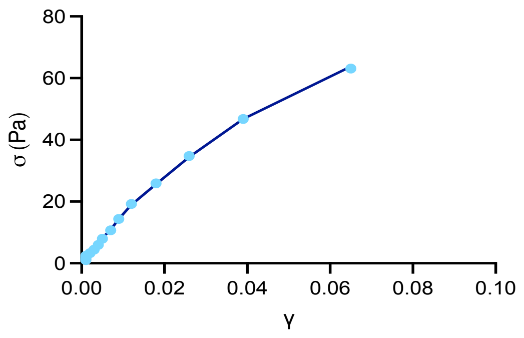

Figure S7. Experimental dependence of the stress (σ, Pa) from strain (γ) for GelMA Cold hydrogel (light blue dots). The blue curve is the best fit of the experimental data using eq. S4. The best-fit procedure is performed using *b* and *G* as floating parameters and minimizing the sum of the square relative error for each experimental data point.

Appendix 3. Rubber elasticity theory for the calculation of the mesh size ($\bar{\xi}$) and the contour length (L_C) of GelMA Hot and Cold hydrogels.

GelMA Hot and Cold hydrogels were investigated according to the rubber elasticity theory. The hydrogels networks were described as a regular mesh with an average mesh size, $\bar{\xi}$, equal to:

$$\bar{\xi} = \sqrt[3]{\frac{6}{\pi \rho N_a}}; \qquad \text{eq. S9}$$

in which ρ describes the network connectivity. We then defined the critical energy, E_c, as the energy required to deform the network and reach the critical deformation (γ_c) (calculated as described in Appendix 2), and is determined via eq. S10 :

$$E_c = \frac{1}{3} k_b T \beta^2 \xi^2 (\lambda^* + \frac{2}{\lambda^*}) \qquad \text{eq. S10}$$

with:

$$\beta^2 = \frac{3}{2L_C(2l_P)}; \text{ and } \lambda^* = \gamma_c + 1 \qquad \text{eq. S11}$$

where L_C is the contour length, l_p is the persistence length and λ^* is the critical stretch ratio. We employ eqs. S10 and S11 to determine the L_c under the assumption that the crosslinks in the GelMA networks follow a Gaussian distribution. The same l_p value is used for Cold and Hot hydrogels, reflecting the supposed random coil (2.0 nm) [5–7]. The results from the calculations are reported in the Table S1 below.

GelMA Hydrogel	$\bar{\xi}$ (nm)	L_c (nm)	N_K
**Cold**	15.7	26	7
**Hot**	25.5	80	20

Table S1. Mesh size ($\bar{\xi}$), contour length (L_c) and number of Kuhn's segments (N_K with $L_C = N_K l_K = N_K(2l_p)$) of GelMA Hot and Cold hydrogels.

Bibliography of Supporting Information:

(1) Lee, H. P.; Gu, L.; Mooney, D. J.; Levenston, M. E.; Chaudhuri, O. Mechanical Confinement Regulates Cartilage Matrix Formation by Chondrocytes. *Nat Mater* **2017**, *16* (12), 1243. https://doi.org/10.1038/NMAT4993.

(2) Turco, G.; Donati, I.; Grassi, M.; Marchioli, G.; Lapasin, R.; Paoletti, S. Mechanical Spectroscopy and Relaxometry on Alginate Hydrogels: A Comparative Analysis for Structural Characterization and Network Mesh Size Determination. *Biomacromolecules* **2011**, *12* (4), 1272–1282. https://doi.org/10.1021/BM101556M.

(3) Sacco, P.; Cok, M.; Asaro, F.; Paoletti, S.; Donati, I. The Role Played by the Molecular Weight and Acetylation Degree in Modulating the Stiffness and Elasticity of Chitosan Gels. *Carbohydr Polym* **2018**, *196*, 405–413. https://doi.org/10.1016/J.CARBPOL.2018.05.060.

(4) Marsich, E.; Travan, A.; Feresini, M.; Lapasin, R.; Paoletti, S.; Donati, I. Polysaccharide-Based Polyanion–Polycation–Polyanion Ternary Systems in the Concentrated Regime and Hydrogel Form. *Macromol Chem Phys* **2013**, *214* (12), 1309–1320. https://doi.org/10.1002/MACP.201300057.

(5) Djabourov, M.; Lechaire, J. P.; Gaill, F. Structure and Rheology of Gelatin and Collagen Gels. In *Biorheology*; IOS Press, 1993; Vol. 30, pp 191–205. https://doi.org/10.3233/BIR-1993-303-405.

(6) Courty, S.; Gornall, J. L.; Terentjev, E. M. Mechanically Induced Helix-Coil Transition in Biopolymer Networks. *Biophys J* **2006**, *90* (3), 1019–1027. https://doi.org/10.1529/biophysj.105.067090.

(7) Grosberg, A. Yu.; Khokhlov, A. R.; Stanley, H. E.; Mallinckrodt, A. J.; McKay, S. Statistical Physics of Macromolecules. *Computers in Physics* **1995**, *9* (2), 171. https://doi.org/10.1063/1.4823390.
